# The Perspective of Forensic Inpatients With Psychotic Disorders on Protective Factors Against Risk of Violent Behavior

**DOI:** 10.3389/fpsyt.2020.575529

**Published:** 2020-11-05

**Authors:** Hiroko Kashiwagi, Yuji Yamada, Yayoi Umegaki, Koji Takeda, Naotsugu Hirabayashi

**Affiliations:** Department of Forensic Psychiatry, National Center of Neurology and Psychiatry, National Center Hospital, Tokyo, Japan

**Keywords:** violence risk, SAPROF, protective factor, psychotic disorder, MTSA, client perspective, self-assessment

## Abstract

**Background:** Little is known about the opinions of forensic inpatients with psychotic disorders like schizophrenia on factors likely to prevent or decrease future violent behavior.

**Aims:** To understand the perspectives of forensic inpatients with psychotic disorders on protective factors against risk of violent behavior and compare them to factors identified by professional staff.

**Method:** Using the Structured Assessment of Protective Factors (SAPROF) checklist for self-appraisal of violence risk, we conducted semi-structured interviews with 32 inpatients of the Medical Treatment and Supervision Act Ward and compared the results with those of professionals.

**Results:** Inpatients scored higher in the SAPROF total score, the motivational factors of “life goals” and “motivation for treatment,” and the protective level in general. Inpatients scored themselves lower in risk level than professionals. The degree of agreement between service users' and professionals' evaluations was low for all categories except external factors. Inpatients prioritized “life goals,” “self-control,” and “medication” as the top three key strengths currently preventing violent behavior, whereas the professionals selected “life goals” less often. The top three important future goals for preventing future violence selected by inpatients were “work,” “intimate relationships,” and “life goals,” with the former two being selected significantly less often by the professionals.

**Conclusions:** This is the first study to shed light on Japanese forensic inpatients' perspectives about preventing future violent behavior. Despite professionals' underestimation, inpatients viewed themselves as having high motivation for treatment and positive life goals. Inpatients prioritized personal values such as life goals, work, and intimate relationships, whereas professionals prioritized understanding, treating, and observing the disease. Our findings are consistent with past reports on patients' and clinicians' perspectives. Awareness of such gaps in perceptions can help build fruitful therapeutic alliances. We discuss the implications in terms of treatment, how to address the gap therapeutically, and how to design treatment accordingly. Directions for future research are also discussed.

## Introduction

In Japan, legislation to provide specialized treatment to forensic service users with mental disorders, along with the Medical Treatment and Supervision Act (MTSA), have been enforced since 2005, symbolizing the beginning of fully fledged forensic psychiatry (1–5). According to the Medical Treatment and Supervision Act Hospitalization Guidelines, developed by the Ministry of Health, Labor and Welfare, service users are required to seek necessary medical care autonomously and be willing to work proactively on their issues (6).

In risk assessment and management of violent behavior, the Structured Professional Judgment (SPJ) method is of mainstream use worldwide. SPJ has been considered an alternative to unstructured clinical judgment and actuarial assessment and has contributed to decision-making in violence risk assessment (7). However, findings from studies on violence risk assessment have suggested no overall standout scheme; thus, greater attention should be directed toward intervention-focused research, as the ultimate purpose of risk assessment is to reduce risk of violent behavior (8).

In recent years, the importance of involving service users in risk assessment has been noted. Particularly, in the risk assessment and management of violence, it is important to focus on the experience and perspective of service users (9). Through transparency and open discussion, a relationship of trust needs to be formed (10, 11); moreover, risk management needs to be conducted positively, the strengths of the service users should be further developed, and recovery must be emphasized to make risk management more effective (9).

The Structured Assessment of Protective Factors (SAPROF) is an assessment tool focusing on strengths and recovery regarding risk of violent behavior (12). Other risk assessment tools that measure protective factors are the Short-Term Assessment of Risk and Treatability (START) (13) and, for adolescents, the Structured Assessment for Violence Risk in Youth (SAVRY) (14). However, the protective factors in the START may simply be reciprocals of the risk factors, while the protective factors in the SAPROF seem to demonstrate a real protective effect (15, 16). The SAPROF evaluates 17 protective factors, including personal characteristics, environmental aspects, and situations that reduce violence risk (12). The SAPROF is expected to contribute to enabling positive risk management, making it easier for service users and healthcare staff alike to cooperate and increase the motivation of both parties.

Few studies have explored cases in which service users were involved in risk assessment and management (17). Risk assessment and shared care planning using the START was ineffective for preventing recidivism in a cluster randomized controlled trial with forensic psychiatry outpatients (18). However, a more recent study by Simpson et al. presented optimistic results (17). Indeterminately sentenced prisoners and their psychologists were interviewed in a previous study on sharing risk assessments (19). Both parties had “remarkably similar views about the assessment interview,” focusing on “emphasizing clarity and transparency,” “collaborative engagement,” “making a respectful and restrained human connection,” “respecting individuality,” and “having a purposeful conversation” (19). These findings highlight the importance of mutual respect between the service user and health care professional in the development of a cooperative therapeutic alliance where both parties share the same goals.

A previous study examined self-assessment of risk and protective factors against violent or criminal behavior with forensic psychiatry outpatients, using START (20); the agreement between the client and case manager on the client's key risk and protective factors was found to be poor (20). According to the predictive validity of client and case manager risk assessment for incidents of violent or criminal behavior in the following 6 months, the area under the curve (AUC) of client mean critical vulnerabilities and client mean key strengths was 0.62 and 0.65, respectively, while those of case managers were 0.53 and 0.54, respectively (20). The authors concluded that risk assessment by the client is feasible, and the optimal prediction model for violent or criminal behavior consisted of the case manager's structured professional risk assessment in combination with the client's self-appraisal on key risk and protective factors (AUC = 0.70) (20).

There have been no previous reports shedding light on the perspective of service users by interviewing them directly about protective factors they believe will reduce the risk of violent behavior and comparing their perspectives to those of professionals. The purpose of this study was to clarify the difference between service users' and professionals' evaluations, and to explore service users' perspectives on protective factors against risk violent behavior.

## Materials and Methods

### Ethical Considerations

All procedures contributing to this work comply with the ethical standards of the relevant national and institutional committees on human experimentation, and with the Helsinki Declaration of 1975, as revised in 2008. All procedures involving human subjects were approved by the Ethical Review Board of the National Center of Neurology and Psychiatry (approval number A2018-087, approved on March 16, 2019). Written informed consent was obtained from all participants after they were given a complete description of the study. This study forms part of a research project registered in the Clinical Trial Registry of the University Hospital Medical Information Network (UMIN; ID: UMIN000035429).

### Instrument

The SAPROF is a checklist of 17 protective factors (Table 1), all of which are rated on a 3-point scale (0 = *the protective factor is clearly absent or there is no evidence that the protective factor is present;* 1 = *the protective factor may be present or is present to some extent*; 2 = *the protective factor is clearly present*) reflecting the extent to which they are present for a given service user in a specific situation (12). The SAPROF items are organized into three scales: internal, motivational, and external factors. Items 1 and 2 (internal factors) are considered static, whereas the other 15 factors are dynamic and therefore changeable during treatment. The total SAPROF protection score is the sum of the scores of the 17 items and ranges from 0 to 34. The total SAPROF internal, motivational, and external scores are the sums of the five (items 1–5), seven (items 6–12), and five (items 13–17) item scores related to these factors, respectively. After rating all the protective factors, a final protection evaluation score, reflecting the degree of protection against relapse into violent behavior, is assigned on a 5-point scale (*low, low-moderate, moderate, moderate-high, high*).

**Table 1:** Structured Assessment of Protective Factors (SAPROF-17) factors and expected changes during treatment.

	**Expected changes during treatment**
**Internal factors**	
1. Intelligence	Static
2. Secure attachment in childhood	Static
3. Empathy	Improving
4. Coping	Improving
5. Self-control	Improving
**Motivational factors**	
6. Work	Improving
7. Leisure activities	Improving
8. Financial management	Improving
9. Motivation for treatment	Improving
10. Attitudes toward authority	Improving
11. Life goals	Improving
12. Medication	Improving
**External factors**	
13. Social network	Improving
14. Intimate relationship	Improving
15. Professional care	Decreasing
16. Living circumstances	Decreasing
17. External control	Decreasing

*Item 14, “Intimate relationship,” refers to marriage and love. Item 16, “Living circumstances,” refers to whether the living environment is supervised by someone*.

The Japanese version of SAPROF (21) was prepared by the authors, and its reliability and prediction validity have been confirmed (22). During the translation process, the original English version was translated into Japanese by the first author (HK), the last author (NH), and six others. Next, back-translation was performed by a native English speaker whose second language was Japanese. Finally, the back-translated version of the SAPROF was confirmed and approved by the Dutch researchers who originally developed the SAPROF.

Within 2 weeks before conducting service user interviews, the SAPROF was evaluated by a professional; those results were considered the professional perspective. As explained above, each of the 17 SAPROF items was scored 0, 1, or 2, and a final overall risk and protection evaluation score was assigned on a 5-point scale. Finally, the three most important or key strengths that helped service users prevent violent behavior at the time of assessment were selected, as well as the three most important future goals to strive for. The SAPROF was completed based on a psychiatric evaluation report compiled by a psychiatrist, a life and environmental report compiled by a probation officer, and clinical records from multi-disciplinary professionals. The SAPROF was scored by the first author (HK), a forensic psychiatrist who attended English and Japanese training sessions for scoring the measure.

A semi-structured interview using the self-evaluation interview version of the SAPROF (23) was conducted with each participant, and the results were considered to represent the service users' perspective. Finally, we asked the participants to select their three most important strengths that helped them prevent violent behavior at the time of assessment, as well as their three most important future goals to reduce risk of violent behavior.

Participants were assessed using the Positive and Negative Syndrome Scales (PANSS) (24) to evaluate psychiatric symptoms (positive and negative symptoms, and general psychopathology) at the time of the SAPROF self-assessment interview by a clinical psychologist (YU).

### Participants

Participants were men and women hospitalized in the National Center Hospital of Neurology and Psychiatry Medical Observation Law Ward (66 beds) in April 2019, who agreed to participate and met the following criteria: participants had to be (1) between the ages of 20 and 70 at the time of consent, and (2) hospitalized for at least the previous month. Service users were excluded from this study if they were (1) diagnosed with at least moderate intellectual disability, (2) diagnosed with dementia, (3) in an at least moderate depressive state or experiencing suicidal thoughts (because the interview could worsen the suicidal thoughts or depressive state), or (4) rejected for participation by the multi-disciplinary team in charge of this study. All participants were taking antipsychotic medications, and their compliance was confirmed by nurses. Inpatients in the MTSA wards are treated by a multi-disciplinary team consisting of a psychiatrist, primary and associate nurses, an occupational therapist, a clinical psychologist, and a social worker. The primary nurse in charge usually manages the case during hospitalization.

### Statistical Analysis

The mean for each of the 17 individual SAPROF item scores, the total score, and the internal, motivational, and external factor scores, as well as the overall risk and protection judgment scores, were compared between service users and professionals via t-test. When the Kolmogorov-Smirnov normality test did not indicate a normal distribution, the Mann-Whitney *U*-test was applied. *P*-values were adjusted with the Bonferroni correction (*p* < 0.0025) for multiple comparisons with 20 items. In addition, the degree of accordance between evaluators (professionals and participants) was analyzed using the intraclass correlation coefficient (ICC) (25). The critical values used for ICCs (single measure) were as follows: ICC ≥ 0.75 = excellent; 0.60 ≤ ICC < 0.75 = good; 0.40 ≤ ICC < 0.60 = moderate; ICC < 0.40 = poor (26).

We also asked the evaluators to pick the three most important currently present strengths and the three most important future goals that helped prevent violent behavior and compared the differences between service users and professionals using Fisher's exact test because of the small sample size. All statistical analyses were performed using IBM SPSS Statistics version 24.0.

## Results

Of the 61 inpatients as of April 10, 2019, 54 had been hospitalized for at least 1 month and were between 20 and 70 years old. Two service users with moderate intellectual disability were excluded. Six service users with suicidal thoughts or at least moderate depression were excluded. The participation of seven service users was rejected by the multi-disciplinary team in charge of the study because their participation would have likely been difficult: three refused treatment or were highly suspicious of the multi-disciplinary team and could not establish a cooperative treatment alliance, one showed severely obsessive behavior (confirmation behavior), and three suffered from significant unrest such as delirium and psychomotor arousal. Another seven service users did not agree to participate in the study. Finally, a total of 32 service users participated in the study. As shown in Table 2, their average age was 41 years; men accounted for three quarters of participants; 87.5% of participants had schizophrenia and all were hospitalized due to psychotic symptoms (the diagnostic categories based on ICD-10 codes were F0, associated with epileptic psychosis, and F1, associated with psychotic symptoms). Nearly 80% of participants had a history of committing murder or assault. The length of hospitalization varied, as shown in Table 2.

**Table 2:** Participant demographic characteristics.

***N* = 32**		
**Age (years)**	**Mean age (SD)**	41.41 (10.45)
Gender	Male	24 (75%)
	Female	8 (25%)
Diagnosis	F00-09	1 (3.1%)
	F10-19	3 (9.4%)
	F20-29	28 (87.5%)
	Paranoid schizophrenia	23
	Undifferentiated schizophrenia	2
	Delusional disorder	2
	Schizoaffective disorder	1
Index offense	Murder	14 (43.8%)
	Bodily injury	11 (34.4%)
	Arson	5 (15.6%)
	Robbery	2 (6.3%)
PANSS	Positive symptoms: Mean (SD)	16.4 (6.6)
	Negative symptoms	18.9 (6.0)
	General psychopathology	37.7 (10.0)
	Total score	73.0 (20.0)
Medication	CPZ eq. (mg/day): Mean (SD)	687.5 (378.7)
	LAI	7 (21.9%)
	Clozapine	10 (31.3%)
Admission duration	Median	425 days (52-1,539 days)
	Mean (SD)	530.3 days (393.8 days)
Stage	Acute	1 (3.1%)
	Recovery	16 (50.0%)
	Rehabilitation	15 (46.9%)

*Diagnostic categories are based on ICD-10 codes as follows: F00–09 (organic, including symptomatic, mental disorders); F10–19 (mental and behavioral disorders due to psychoactive substance use); F20–29 (schizophrenia, schizotypal, and delusional disorders). The acute stage refers to being within 3 months of hospitalization, or having acute or subacute psychological symptoms, and not having recovered the basic judgment ability. The recovery stage is a state where acute or subacute psychological symptoms resolve, basic judgment ability improves, and it is possible for the patient to adapt to the ward and go out. The rehabilitation stage allows patients to stay overnight to prepare for discharge, in addition to the recovery stage features. PANSS, Positive and Negative Symptom Scale; LAI, Long Acting Injection; CPZ, Chlorpromazine; eq, equivalent*.

Table 3 shows the comparison of mean evaluation scores between service users and professionals. Compared with professionals' evaluations, service users showed higher overall and motivational factors scores. They judged protection levels to be high—meaning that their ability to inhibit violent behavior was strong—and evaluated risk levels as low. As for individual items, service users scored higher on “motivation for treatment” and “life goals” than professionals scored them, meaning that they perceived themselves as having stronger goals in life and higher motivation to go through with treatment, compared to how they were perceived by the professionals.

**Table 3:** Comparison between service user and professional assessments.

**SAPROF**	**Service user assessment Mean (SD)**	**Professional assessment Mean (SD)**	***P*-value**	**95% CIs**
Total score	21.91 (3.98)	18.63 (3.85)	**0.001**	1.33–5.24
Internal factors	5.56 (1.48)	4.50 (1.59)	0.004	0.30–1.83
Motivational factors	9.00 (2.31)	7.00 (2.08)	**0.001**	0.90–3.10
External factors	7.34 (1.23)	7.06 (0.91)	0.25	−0.26–0.83
Protection level assessment	3.94 (0.98)	2.81 (0.93)	**<0.001**	0.65–1.60
Risk level assessment	1.72 (0.89)	2.56 (0.80)	**<0.001**	−1.27−-0.42
SAPROF individual items				
Intelligence	0.97 (0.70)	0.50 (0.57)	0.006	0.15–0.79
Secure attachment during childhood	1.22 (0.75)	1.25 (0.80)	0.811	−0.42–0.36
Empathy	1.13 (0.49)	0.94 (0.44)	0.109	−0.045–0.42
Coping	1.06 (0.56)	0.81 (0.40)	0.051	0.006–0.49
Self–control	1.19 (0.47)	1.00 (0.67)	0.230	−0.10–0.48
Work	0.66 (0.79)	0.56 (0.50)	0.929	−0.24–0.42
Leisure activities	0.84 (0.77)	0.84 (0.45)	0.818	−0.32–0.32
Financial management	1.50 (0.57)	1.34 (0.65)	0.354	−0.15–0.46
Motivation for treatment	1.72 (0.52)	1.09 (0.53)	**<0.001**	0.36–0.89
Attitude toward authority	1.53 (0.57)	1.16 (0.52)	0.006	0.10–0.65
Life goal	1.41 (0.76)	0.75 (0.44)	**<0.001**	0.35–0.97
Medication	1.34 (0.60)	1.25 (0.44)	0.369	−0.17–0.36
Social network	1.13 (0.75)	0.88 (0.49)	0.120	−0.068–0.57
Intimate relationship	0.34 (0.70)	0.19 (0.54)	0.316	−0.16–0.47

*T-test only for total score and motivational factors, Mann-Whitney U-Test for all other factors. 95% confidence intervals (CIs) following t-test are shown. P-value: Bonferroni-adjusted (p < 0.0025)*.

Table 4 shows the level of agreement between professional and service user evaluations. “Intimate relationship” and “external factors” showed high levels of agreement, but there was little agreement on the other items.

**Table 4:** Level of agreement between service user and professional assessments.

**SAPROF**	**ICC**	**P-value**
Total score	0.19	0.078
Internal factors	0.21	0.072
Motivational factors	0.12	0.187
External factors	**0.49**	0.001
Protection level assessment	−0.105	0.743
Risk level assessment	0.032	0.424
SAPROF individual items		
Intelligence	0.03	0.412
Secure attachment in childhood	0.29	0.057
Empathy	0.18	0.149
Coping	0.046	0.389
Self–control	0.37	0.014
Work	0.014	0.470
Leisure activities	0.27	0.069
Financial management	0.21	0.114
Motivation for treatment	0.06	0.293
Attitude toward authority	0.30	0.018
Life goals	0.12	0.145
Medication	0.15	0.21
Social network	0.27	0.057
Intimate relationship	**0.65**	<0.001

*Intraclass correlation coefficient (ICC) ≥ 0.75 = excellent; 0.60 ≤ ICC < 0.75 = good; 0.40 ≤ ICC < 0.60 = moderate. The bold values mean ICC > 0.40*.

**Figure 1:**
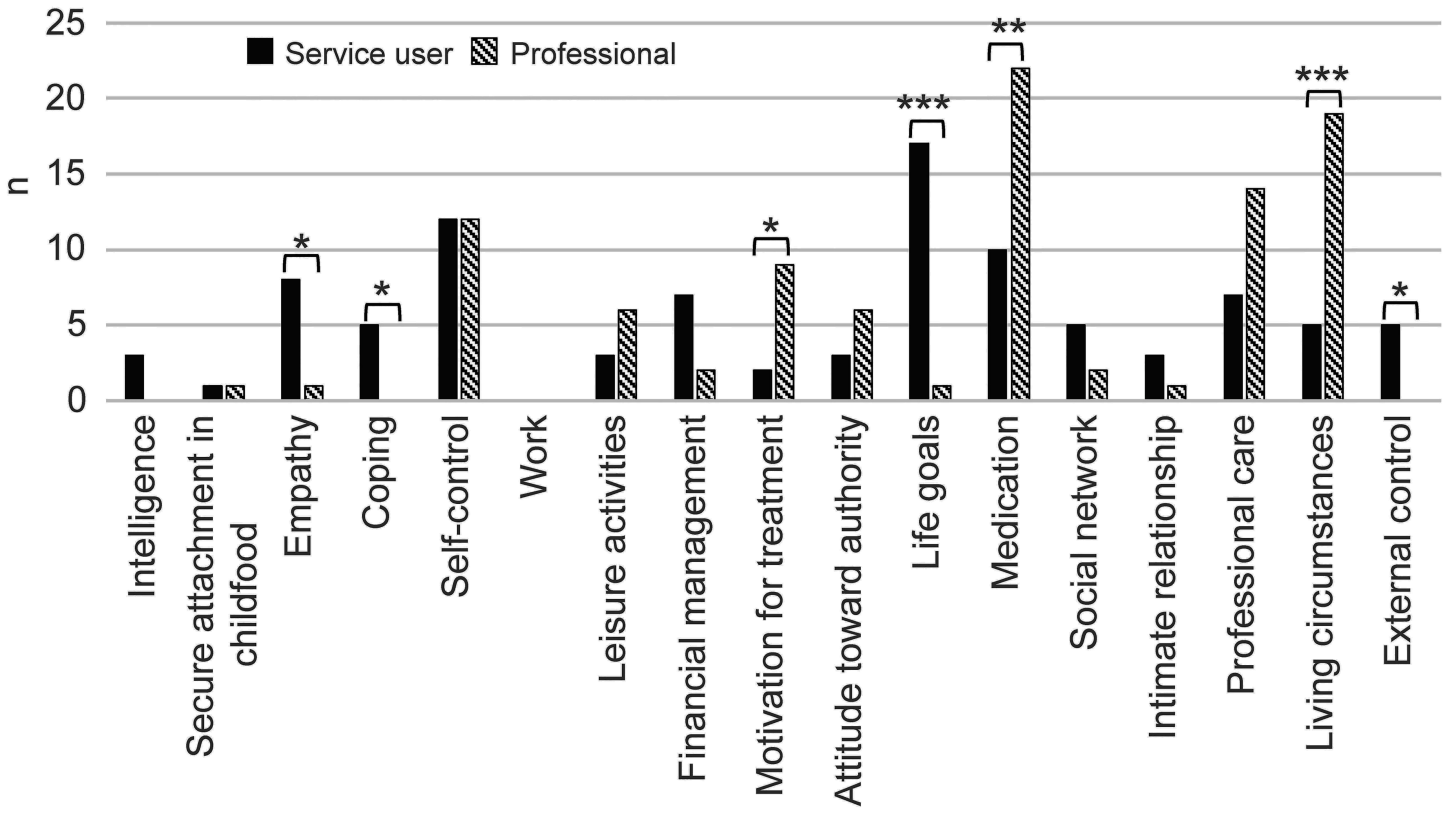
Current key strengths for violence prevention: service users compared with professionals. Patients and professionals selected three key protective factors. The number of persons who selected each item is shown in the bar graph. The rates of selection were compared using Fisher's exact test. Fisher's exact test: *p < 0.05; **p < 0.01; ***p < 0.001.

We had participants select the three most important current key strengths that helped prevent violent behavior. Figure 1 displays the number of participants selecting each item and the comparison between service users' and professionals' responses. Participants most often chose “life goals,” followed by “self-control” and “medication.” “Life goals” was selected particularly often by service users and rarely by professionals, leading to a significant difference in selection. Further, the number of participants selecting “empathy,” “coping,” and “external supervision” was proportionally significantly higher than that among professionals, indicating a difference in perspectives. Of the top three items selected by professionals, the first was “medication,” followed by “living circumstances” and “professional care.” The number of professionals who selected “living circumstances,” “medication,” and “motivation for treatment” was proportionally significantly higher than that among service users, also indicating a difference in perspectives.

**Figure 2:**
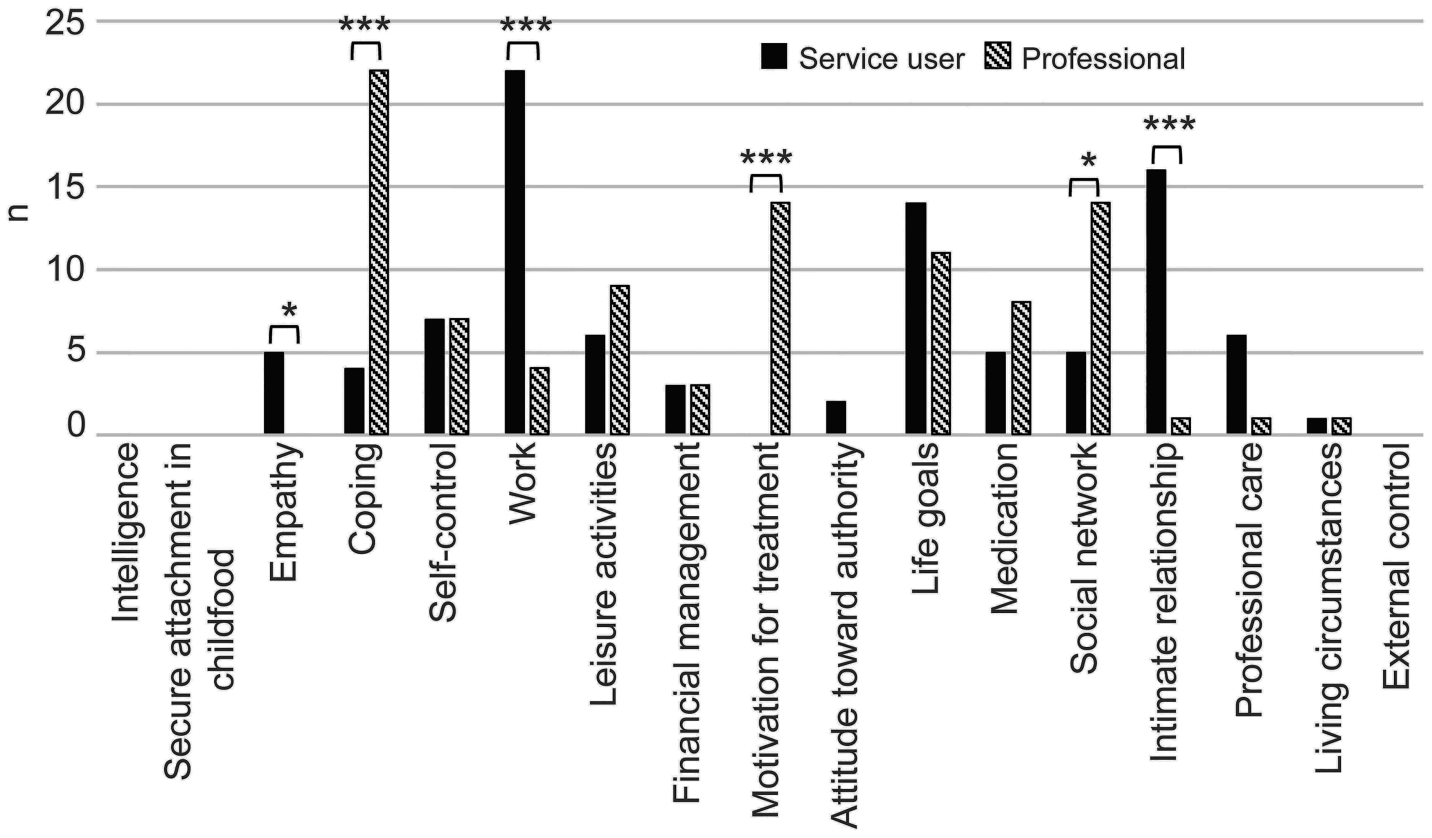
Goals to prevent violence risk: services users compared with professionals. Patients and professionals selected three protective factors each as future goals. The number of persons who selected each item is shown in the bar graph. The rates of selection were compared by Fisher's exact test. Fisher's exact test: *p < 0.05; **p < 0.01; ***p < 0.001.

As shown in Figure 2, we asked participants to select the three future goals they perceived as most important in helping prevent violence risk. Figure 2 shows the number of participants selecting each item and the comparison between service users' and professionals' responses. Service users most often chose “work,” “intimate relationships” (marriage and love), and “life goals.” “Work” and “intimate relationships” were selected particularly often among participants in comparison with professionals, indicating yet another difference in viewpoints between the two groups. “Empathy” was also selected more often by service users. The top choice of professionals was “coping,” followed by “motivation for treatment” and “social network.” All three items were selected significantly more often by professionals than by service users, indicating a clear difference in viewpoints.

## Discussion

To the best of our knowledge, this is the first study to shed light on Japanese forensic inpatients' perspectives about preventing violence risk. We interviewed forensic inpatients to evaluate what protective factors might lower the risk of violent behavior, enquiring about their current strengths, what they currently found most useful for preventing violent behavior, and what their most important goals were. We then compared these results with the evaluations of professionals.

Service users and professional assessments tended to agree on external factors but not on the others. Compared with professionals, inpatients evaluated the overall level of factors' protection as high and risk as low, giving “life goals” and “motivation for treatment” particularly high scores.

Higher evaluations of these recovery-related factors by psychotic patients compared with professionals is a consistent finding, also observed in non-forensic samples (27). Previous findings have indicated that staff rate service users as having higher needs than indicated by the service users themselves in non-forensic samples (27). Additionally, the forensic version of the Camberwell Assessment of Need (CANFOR) found a gap between the perceived needs of patients as assessed by clinicians and patients themselves (28, 29). Forensic patients reported significantly fewer needs, whether met or unmet, than did their treating clinicians (28), which is consistent with findings from non-forensic patients.

Regarding social quality of life, clinicians have been reported to estimate clients' social quality of life as being poorer than clients' own estimations when the clients had low social cognition and severe negative symptoms (30). Regarding motivation for treatment, it has been reported that clinicians showed poor to moderate ability to estimate patients' perspectives on their motivation for engaging in treatment (31). Our findings suggested that clinicians might estimate clients' levels of life goals and motivation for treatment as being poorer than clients' own estimates if the clients had a history of severe offense and severe mental illness. Therefore, interviewing clients for perspectives on their life goals and motivation for treatment must be important longitudinally, particularly in forensic settings.

While service users valued “life goals,” “work,” and “intimate relationships” (marriage and love), professionals tended to focus more on items related to understanding, observing, and treating disease. In a Danish study of user perspectives on mental health services with 50 offenders with mental disorders (32), those interviewed about the experience of being forensic service users and how to improve services showed remarkable similarities in their answers, stating that it was important for the staff to interact with them with respect and empathy. Moreover, when asked what it was like to be a forensic service user, they answered that they were “first and foremost a human being” (p. 593, 595) (32). In a study on forensic addiction treatment, patients and therapists appeared to have very different views on what happened during treatment and why therapy eventually failed (33). While therapists cited patients' unwillingness to make an effort or to change behavior as the most important reasons for therapy failure, patients highlighted psychological tension and aggressiveness, frequent quarrels with fellow patients, and abysmal therapeutic environment (33). Our study found that forensic service users emphasized the importance of “work,” “life goals,” and “intimate relationships” for the prevention of violence, and that, contrary to professionals' underestimation, service users perceived themselves as having life goals and high treatment motivation. It is natural that service users with such self-understanding in particular would want staff to treat them with respect.

In this study, while professional judgment was more focused on items related to treatment and observation, inpatients rarely selected medical-care-related items for goals for preventing violent behavior and instead leaned toward aspects of their lives outside treatment, such as life goals, work, and love. In an online questionnaire study on the medication adherence of schizophrenic service users, 64% reported having previously stopped their medication on their own accord, and 58% reported needing no antipsychotics (34). The present study found similar results, as service users tended to focus more on their lives outside of treatment.

Davoren et al. reported that self-rated scores for program completion and forensic recovery did not predict moves between levels of therapeutic security or conditional discharge (35), while the level of concordance—the gap between clinician and patient ratings—did predict outcomes. The authors concluded that the means to improve this concordance is of great interest and may in itself be an appropriate outcome measure for various forms of psycho-education and treatment programs (35). The effectiveness of shared risk assessment and management has been inconsistent and requires further research (17, 18).

In our study, differences in perspectives were found between inpatients and professionals about protective factors against violent behavior, which is why it is important for both parties to first learn about each other's viewpoints through dialogue. When judicial psychiatry stalls, it may be necessary to bring in such new perspectives and introduce new treatment techniques. One way to build a good therapeutic alliance with service users who hold different perspectives may be to promote mutual self-reflection through expressing viewpoints in open dialogue (36, 37). Such self-reflection may encourage service users' self-initiative.

Urheim et al. reported that an increase in individualized, patient-oriented care strategies, delivered with an equally balanced gender distribution by well-educated nursing staff, contributed to a low level of violent behavior (38). While many forensic service users consider work to be the most important goal for the prevention of violent behavior, only 13.8% of service users who had left medical treatment and supervision wards in Japan have been able to find employment, with full-time employees accounting for only 5.4% of the examined sample (39). The employment rate is also not high for service users discharged from overseas forensic psychiatric wards (39). Nevertheless, work is a protective factor against the risk of violent behavior (12), and individual placement and support are known to increase employment rate and improve the prognosis of service users with severe disorders, such as schizophrenia (40, 41). Verifying the effect of individual placement and support on offenders with mental disorders needs to be the focus of future studies (42).

Our results suggest that clinicians might tend to underestimate the patient's own perspective on life goals and treatment motivations. Furthermore, it was found that what patients consider to be their current strengths as a protective factor for the risk of violence and what they want to value as future goals as a protective factor for the risk of violence are different from the clinicians' assessments. Especially in the setting of forensic psychiatry, clinicians should continue to interview patients on their own perspectives on life goals and treatment motivations, as well as patients' own perspectives on their strengths and goals longitudinally in order to build a good therapeutic alliance. Finally, treating service users who regard themselves as having life goals and motivation for treatment with respect, and establishing “work,” “intimate relationships,” and “life goals” as common aims, may facilitate the development of a cooperative therapeutic alliance.

### Limitations

First, the number of participants (32) was small and included an uneven distribution of gender (24 of the 32 were male) and diagnostic groups (28 of the 32 were schizophrenic). This small sample size and the fact that it is impossible to know whether the results would have been different with a different gender ratio or different diagnostic groups implies that the present findings should be interpreted with caution. Second, too many comparisons for a sample of this size raises the possibility of Type 1 error. Third, only a single facility was involved, the sample was limited to individuals who agreed to participate during their hospitalization, and the evaluated treatment stages were not constant. The idea that patients who are forced to be hospitalized want to be discharged as soon as possible may explain the differences between self-assessment and professional ratings of risk level and treatment motivation. Given these limitations, generalization is difficult. Last, professional evaluations tended to stem from a short-term perspective, while service users' perspectives tended to be long-term-oriented. These differences in temporal orientation between professionals and service users may result in professionals tending to focus more on clinical items and service users focusing more on life events.

### Further Research

We plan to conduct further research with a larger number of participants and multiple facilities in the future. Studies on comparisons between the perspectives of male and female patients, between diagnostic categories, and between types of crime would be the focus of future research, so as to develop a more in-depth understanding. The value of the current study is limited by the lack of follow-up to determine which prediction—that of service users or professionals—was more accurate. While this is obviously required for the validation of a risk assessment instrument, forensic psychiatry has moved on to structured professional judgment of other processes and outcomes, particularly concerning treatment completion, unmet needs, and recovery in a forensic context. Therefore, further research could compare professional-led, service-user-led, and collaborative risk assessments in terms of the predictability of violence; the relationship between the likelihood of violence risk and the level of agreement with professional evaluations; and the effectiveness of violence prevention in service-user-led risk assessment and management.

## Data Availability Statement

The raw data supporting the conclusions of this article will be made available by the authors, without undue reservation.

## Ethics Statement

The studies involving human participants were reviewed and approved by The Ethical Review Board of the National Center of Neurology and Psychiatry (approval number A2018-087). The patients/participants provided their written informed consent to participate in this study.

## Author Contributions

HK contributed to scoring the SAPROF, interviewing participants, and drafting the manuscript. YU assessed the PANSS for all participants. HK and KT contributed to the statistical analysis. HK, KT, YY, YU, and NH contributed to the design and management of the study. All authors approved the final version of the manuscript.

## Conflict of Interest

The authors declare that the research was conducted in the absence of any commercial or financial relationships that could be construed as a potential conflict of interest.
